# First report of the sting nematode *Belonolaimus longicaudatus* infecting bermudagrass in Barbados

**DOI:** 10.21307/jofnem-2020-021

**Published:** 2020-03-30

**Authors:** P. Mc Groary, W. Ye, E. Nangle

**Affiliations:** 1 Waypoint Analytical, Richmond, USA; 2 Nematode Assay Section, Agronomic Division, North Carolina Department of Agriculture & Consumer Services, Raleigh, NC; 3 The Ohio State University, ATI/OARDC, Wooster, OH

**Keywords:** *Belonolaimus longicaudatus*, Barbados, Bermudagrass, First report

## Abstract

In 2016, “Tifdwarf” hybrid bermudagrass (*Cynodon dactylon* (L) Pers. × C. *transvaalensis* Burtt-Davy) grown on a golf green built to the United States Golf Association recommendations in Barbados started to show irregular significant chlorotic patches followed by gradual thinning and decline of turfgrass. A survey was conducted in May 2016 to determine the presence of plant-parasitic nematodes. The results revealed the presence of the plant-parasitic sting nematode *Belonolaimus longicaudatus*. To our knowledge, this is the first report of *B. longicaudatus* associated with bermudagrass in Barbados.

In 2016, “Tifdwarf” hybrid bermudagrass (*Cynodon dactylon* (L) Pers. × *C. transvaalensis* Burtt-Davy) grown on a golf green built to the United States Golf Association recommendations in Barbados started to show irregular significant chlorotic patches followed by gradual thinning and decline of turfgrass. Additionally, turfgrass roots sampled from the symptomatic patches appeared to be abbreviated compared to non-symptomatic areas of the greens. A survey was conducted in May 2016 to determine the presence of plant-parasitic nematodes. Four different soil samples were collected from the edge of the symptomatic patches in four greens by collecting 10 cores to a depth of 15 cm using a 0.5 cm diameter soil probe. Subsequently, soil samples were extracted using decanting and sugar centrifugal flotation technique (Jenkins, 1964). The results revealed the presence of the plant-parasitic sting nematode *Belonolaimus longicaudatus*. The population densities of the four greens were 35, 40, 80 and 90 sting nematodes/100 cm^3^ soil. Morphological and molecular analyses were conducted to determine the species. Female (*n* = 10): L = 2,354.5 ± 94.3 (2,140.0-2,552.0) μm, a = 49.5 ± 7.4 (45.6-58.2), b = 7.2 ± 0.7 (6.8-8.5), c = 17.9 ± 1.5 (15.6-19.7), c’ = 3.6 ± 0.5 (3.5-4.1), V = 51.2 ± 1.2 (51.5-52.8), stylet length = 127.9 ± 5.5 (120.2–139.2) μm, tail length  =  133.3 ± 15.8 (108.7–157.3) μm, excretory pore from anterior end  =  245.8 ± 13.2 (240.7–285.0) μm. Male (*n* = 8): L = 1,945.3 ± 20.3 (1,935.0–1,976.0) μm, a = 51.3 ± 1.3 (49.5–51.3), b = 7.5 ± 0.9 (6.5–8.1), c = 13.5 ± 0.7 (12.8–13.2), c’ = 5.5 ± 0.5 (5.3–6.1), stylet length = 117.0 ± 1.5 (119.0–118.0) μm, spicule length = 54.3 ± 1.5 (52.3–55.4) μm, tail length = 152.3 ± 3.5 (142.3–154.5) μm, excretory pore from anterior end = 223.5 ± 3.5 (221.0–228.0) μm. These measurements are in agreement with *B. longicaudatus* (Rau, 1958). PCR and DNA sequencing on ribosomal DNA 18 S and ITS were performed and the 2,300-bp sequence was deposited in GenBank under the accession number MH985155. BLAST search results revealed it is identical or with a few nucleotide differences with 99 to 100% identity to some populations of *B*. *longicaudatus* in GenBank, but more variable in ITS region with 96 to 99% identity. The molecular data further supported the morphological identification. *B. longicaudatus* was first described from soil around the roots of corn *Zea mays* L. in Sanford, Florida by Rau (1958). It is a major pest in southeastern USA and is widespread throughout the Atlantic coastal plain from Virginia to Florida. Outlier populations have been reported from Mexico and Central America. Reports from Bermuda, the Bahamas and Puerto Rico apparently refer to golf courses where infected turf was imported from the USA (Perry and Rhoades, 1982). In addition to turfgrasses, it also damages soybean (*Glycine max* L. Merr.), cotton (*Gossypium hirsutum* L), corn (*Zea mays* L), strawberry (*Fragaria* x *annanasa* Duch.) and citrus. To our knowledge, this is the first report of *B. longicaudatus* associated with bermudagrass in Barbados.
